# Laparoscopic Management of a 20 cm Ovarian Serous Cystadenoma at 20 Weeks of Pregnancy: A Case Report

**DOI:** 10.7759/cureus.80750

**Published:** 2025-03-18

**Authors:** Ritu Singh, Poonam Lal, Swaroop R Nanda, Rupa Ranee, Avinash K Singh

**Affiliations:** 1 Obstetrics and Gynaecology, Kurji Holy Family Hospital, Patna, Patna, IND; 2 Radiodiagnosis, Indira Gandhi Institute of Medical Sciences, Patna, IND

**Keywords:** huge ovarian tumor, laparoscopic surgery, laparoscopy in pregnancy, ovarian cyst, second trimester pregnancy, serous cystadenoma

## Abstract

Despite adnexal masses being common in the first trimester of pregnancy, large cystic tumours are rare. The management of pregnancy with an ovarian mass is challenging due to the limited data available on the size at which surgery is required. To the best of our knowledge, we did not find any case in the literature of a pregnancy with a 20 cm cyst in the second trimester managed by laparoscopy. Therefore, we present a case of a 20 cm ovarian cyst at 20 weeks of pregnancy.

Our case describes an incidentally diagnosed benign ovarian cyst at 20 weeks of pregnancy in a 24-year-old patient who had previously delivered vaginally. Magnetic resonance imaging (MRI) revealed a cystic lesion measuring 19.8 x 14.8 x 10.6 cm in the left lumbar region, extending into the left iliac fossa, with the left ovary not separately visualized. After a thorough workup, the patient was managed laparoscopically. The patient tolerated the procedure well.

Even in cases of large benign cysts greater than 10 cm, laparoscopy can be safely performed during pregnancy when done by experienced hands with adequate anesthetic facilities, without adverse effects on the fetus. However, proper case selection is mandatory, and further studies are needed before it can be recommended as routine practice.

## Introduction

Despite adnexal masses being common in the first trimester of pregnancy, large cystic tumours are rare [1]. The incidence of large adnexal masses during pregnancy is about 0.2-2%, with the majority being benign [2]. During the first trimester, corpus luteal cysts and theca lutein cysts are most commonly present [1], and they typically resolve by 16 weeks. These masses are generally incidental findings on ultrasound (USG) [1].

Except in cases of acute abdomen (such as torsion), where surgical intervention is necessary regardless of the trimester, the second trimester is considered the best time for surgery [3].

Managing pregnancy with an ovarian mass remains challenging due to limited available data. A review article suggests a general opinion that surgery is required if the cyst size exceeds 10 cm [4], but no specific guidelines exist regarding the size threshold for intervention in asymptomatic patients, as there is always a risk associated with intervention during pregnancy.

There is often a dilemma about whether to perform laparoscopy or laparotomy during pregnancy due to the potential effects of general anaesthesia on the fetus. However, laparoscopy has the advantage over laparotomy of less postoperative pain and a shorter hospital stay. It also has fewer adverse fetal effects in comparison to laparotomy, although this is inconsistent in the literature [4]. In expert hands, laparoscopy during pregnancy is safe, and anaesthetic drugs have not been shown to have teratogenic effects on humans [4].

Large cysts are rare in pregnancy. To the best of our knowledge, we did not find any case in the literature of a pregnancy with a 20 cm cyst in the second trimester managed by laparoscopy. Therefore, we present here a case of a 20 cm ovarian cyst at 20 weeks of pregnancy.

## Case presentation

A 24-year-old married female came for a routine antenatal visit at our hospital at four and a half months of gestation. She had a spontaneous conception and had not undergone any antenatal checkups in any hospital before. She had not taken folic acid or iron-calcium tablets prior to this visit and could not remember the date of her last menstrual period. In the first trimester, she had no history of radiation exposure or teratogenic drug intake.

She had regular menstrual cycles. She had been married for three years and had one spontaneous abortion at two months of gestation, for which dilation and evacuation were performed. She has a two-year-old male child, who was delivered vaginally and weighed 3.5 kg at birth. She had not used any contraception methods. There were no significant past medical or surgical histories.

On examination, her height was 152 cm, weight was 50 kg, with a BMI of 21.6 kg/m^2^. Her general condition was fair, with pallor present, but no edema or dehydration. Her vitals were stable. On respiratory system examination, bilateral air entry was normal, and no added sounds were heard. The cardiovascular examination was normal. On per abdominal examination, inspection revealed a distended abdomen with linea nigra and striae gravidarum. On palpation, the uterus was found to be the size of 20 weeks, with a symphysis-fundal height of 19 cm. A vague cystic mass was felt in the left lumbar and left hypochondrium, but we could not make out any distinct mass. A speculum examination showed that the cervix was pulled up and of a parous type. Bimanual examination revealed a 20-week-sized uterus with bilateral fornices free.

**Figure 1:**
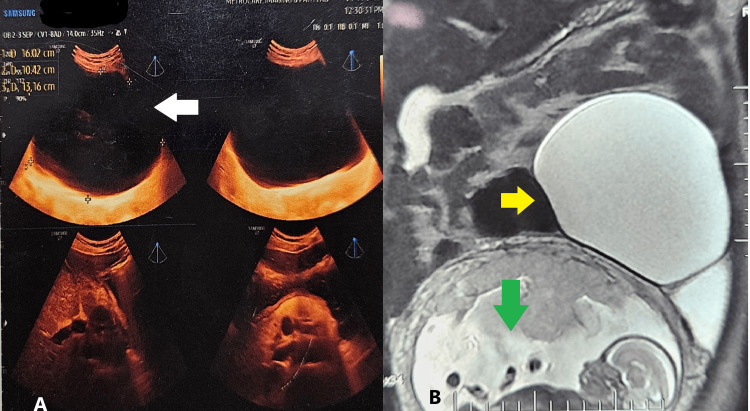
Radiological Investigation (A) Ultrasound: The white arrow points to a benign cystic structure. (B) Magnetic resonance imaging (MRI): The green arrow points to the fetus, while the yellow arrow highlights a hyperintense fluid-filled cystic lesion.

A TIFFA (Targeted Imaging for Fetal Anomalies) USG was done on an outpatient basis and revealed an 18-week pregnancy with a large anechoic cyst measuring 16 x 10 x 13 cm, extending from the epigastrium to the pelvis. The provisional diagnosis was a pancreatic pseudocyst or mesenteric cyst. The USG image is shown in Figure 1A. Since the diagnosis was not clear, magnetic resonance imaging (MRI) was advised. MRI revealed a cystic lesion measuring 19.8 x 14.8 x 10.6 cm in the left lumbar region, extending into the left iliac fossa, with the left ovary not separately visualized. The diagnosis suggested a large left ovarian mucinous cystadenoma, with a differential diagnosis of mesenteric cyst. The MRI film is shown in Figure 1B.

We performed her tumour marker tests, as listed in Table 1, along with the risk of ovarian malignancy algorithm (ROMA), which showed a result of 5.45 (high risk: >7.4%, low risk: ≤7.4%). Serum amylase and lipase levels were also measured to rule out pancreatic pathologies. In other antenatal investigations, all her other parameters were normal, except for haemoglobin, which was 7.1 g/dL.

**Table 1:** Lab Parameters WBC: white blood cells; CA-125: cancer antigen 125; HE4: human epididymis protien 4; AFP: alpha-fetoprotein; LDH: lactate dehydrogenase

Lab Parameters	Lab Values	Reference Values
Haemoglobin	7.1 gm/dL	12-16 gm/dL
WBC	7.5 x 10^3^/µL	5.2-12.4 x 10^3^/µL
CA-125	32.1 U/mL	0-35 U/mL
HE4	42.6 pmol/L	>70 pmol/L
AFP	42.1 ng/mL	10-150 ng/mL
LDH	156 U/L	135-214 U/L
Serum Amylase	44 U/L	30-110 U/L
Lipase	46 U/L	0-160 U/L

With proper consent for cystectomy versus oophorectomy, as well as laparotomy if needed, and after explaining the remote possibility of miscarriage, the patient was planned for diagnostic laparoscopy and the procedure was carried out. In preoperative preparation, two packed red blood cells were transfused to raise her haemoglobin levels. She was also given an intramuscular injection of hydroxyprogesterone 500 mg, and an injection of enoxaparin 40 mg subcutaneously for perioperative thromboprophylaxis, starting two days before the operation and continuing up to 24 hours before the procedure. We started administering dydrogesterone 10 mg twice a day, along with progesterone 200 mg (two capsules) per vaginally at bedtime. Additionally, a single intramuscular injection of 10 mg of Duvadilon was administered 30 minutes before the operation.

**Figure 2:**
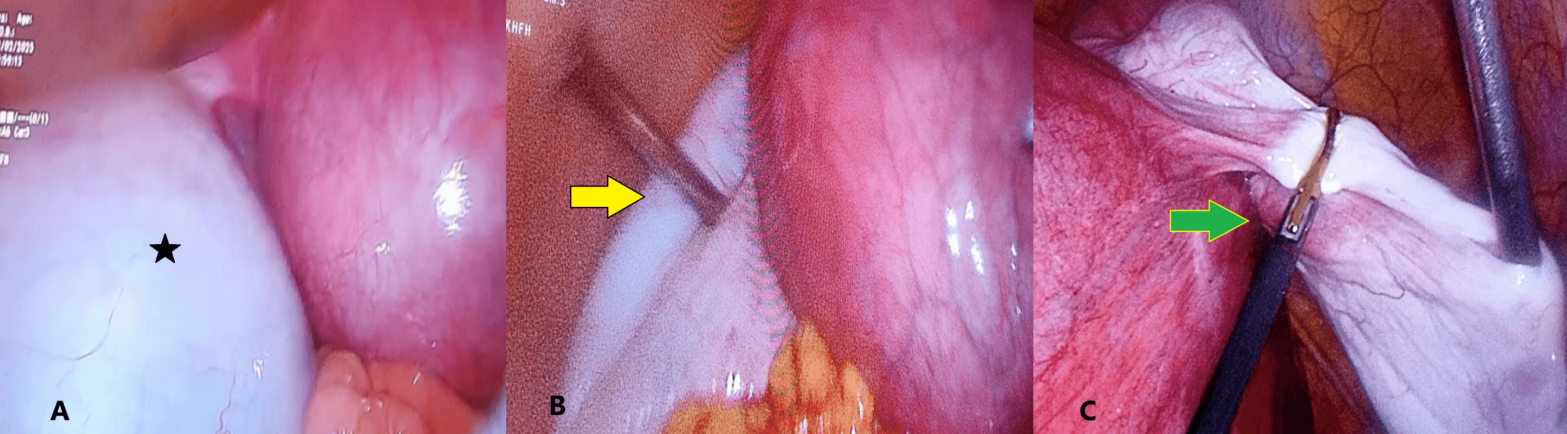
Intraoperative Images (A) The asterisk (*) shows a large ovarian cyst attached to the uterus by the ovarian ligament. (B) The yellow arrow indicates direct trocar entry and aspiration of fluid from the ovarian cyst. (C) The green arrow shows the application of LigaSure (Medtronic, Dublin, Ireland) from the right-side port in front of the uterus.

In the operating room, an intermittent pneumatic compression device was applied to the patient to prevent deep vein thrombosis, with the pressure set at 60 mmHg. After general anaesthesia, cleaning and draping, a Veress needle was inserted 7-8 cm above the uterine fundus, and a 30-degree left lateral tilt was given to the patient (to prevent aortic compression) using a remote-controlled operating table. The gas was then introduced to create pneumoperitoneum. When the pressure reached 12-15 mmHg, the table was immediately tilted from the left lateral to the supine position, and a 10 mm camera port was inserted. After camera insertion, the left lateral tilt was again applied. On diagnostic laparoscopy, although visualization was limited by the gravid uterus, the cyst was seen attached to the uterus by the left ovarian ligament, as shown in Figure 2A.

A 5 mm port was then placed on the right side of the abdomen just above the umbilicus. In the left lateral position, the cyst on the left side was not accessible for direct cannula entry. Therefore, the patient was turned to a 30-degree right lateral tilt, and the left 5 mm port was inserted directly into the cyst. Clear serous cystic fluid was completely drained out by inserting a suction cannula to prevent even minimal spillage, as shown in Figure 2B.

**Figure 3:**
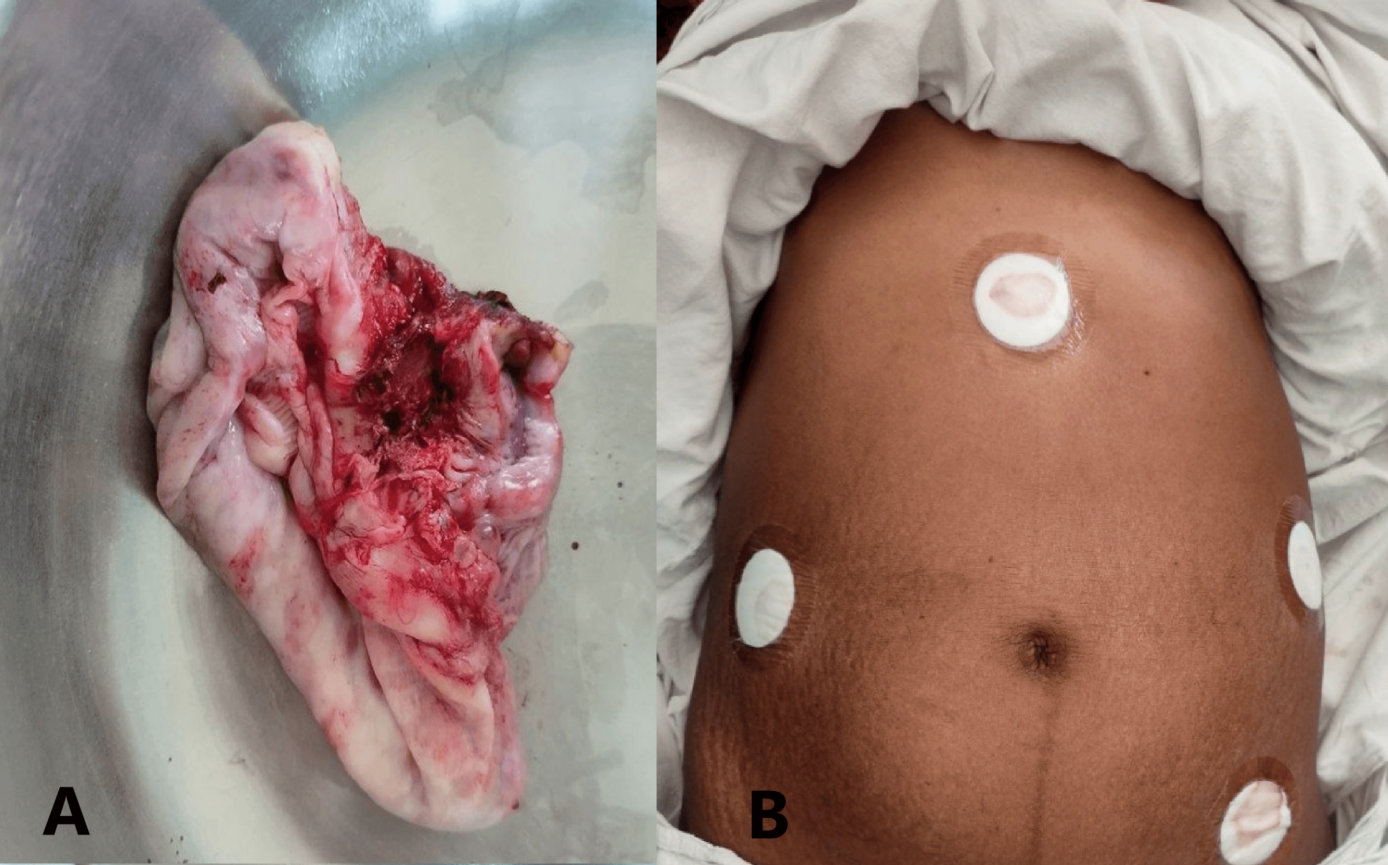
Postoperative Images (A) Retrieved specimen. (B) Port entry sites with the gravid uterus.

Then, the third cannula was inserted two inches above and two inches medial to the right anterior superior iliac spine. Throughout the procedure, intraabdominal pressure was maintained at 12 mmHg. There was difficulty in manipulating the ovary as the entire abdomen was occupied by the gravid uterus. After aspirating the cyst, we were unable to find any ovarian tissue separate from the cyst, so the decision to perform a salpingo-oophorectomy was made. From the left upper port, the ovary and tube were pulled medially, and from the right side port, salpingo-oophorectomy was performed using LigaSure (Medtronic, Dublin, Ireland), as shown in Figure 2C. The sample was removed through the right side 5 mm lower port, which was then enlarged to 10 mm and sent for histopathology. As shown in Figure 3A, the cyst was approximately 20 cm in size, ovoid in shape, with a smooth surface, soft consistency, grey-white colour, and multiloculated. The patient tolerated the procedure well and remained stable throughout the intraoperative period. The fetal heart rate heard on the operating table after the procedure was normal. Her postoperative condition, showing the port sites and gravid uterus, is shown in Figure 3B.

**Figure 4:**
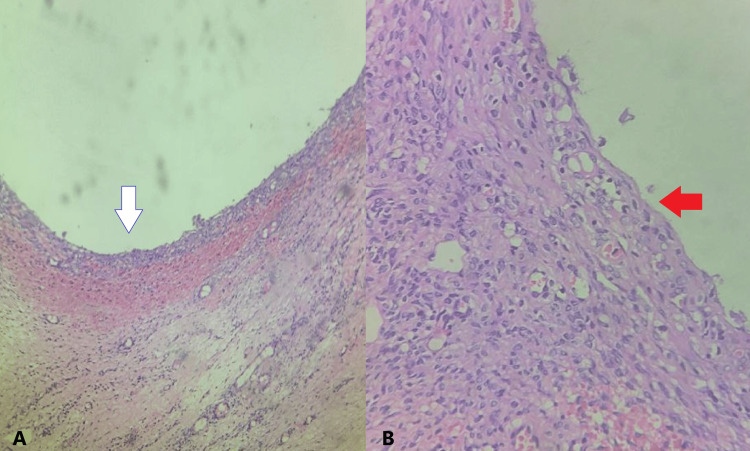
Histopathology Images (A) Histopathology of the specimen (white arrow) showing a thin-walled cyst with epithelial and stromal components (H&E, 10×). (B) Higher magnification displaying low cuboidal epithelium (red arrow) with clear cytoplasm and no atypical cells (H&E, 40×).

The intermittent pneumatic compression device was continued overnight, and then an injection of enoxaparin 40 mg subcutaneously was given daily for three days. Her post-operative period was uneventful, with a post-operative haemoglobin of 10 g/dL. She was discharged on the third day after surgery. Other medications, including hydroxyprogesterone 500 mg injection, dydrogesterone 10 mg tablet, and progesterone 200 mg capsule, were continued. The patient returned for a follow-up after one week and was asymptomatic. Her USG was within normal limits. The histopathology report showed a serous cystadenoma of the ovary, as shown in Figure 4.

## Discussion

Our case was a large ovarian serous cystadenoma. According to a recent review, there is an 11% (range: 4-19%) incidence of ovarian serous cystadenomas in pregnancy requiring surgical management [4]. We had planned the surgery electively, which is supported by a recent meta-analysis by Cagino et al. The authors showed that in elective surgeries, there was a decreased risk of preterm delivery (odds ratio (OR) 0.13; 95% confidence interval (CI): 0.04-0.48; p = 0.05) [5].

Deciding when to perform surgery during pregnancy is a critical decision. According to the American College of Obstetrics and Gynecology (ACOG) committee opinion of 2017, the second trimester is the best time to perform non-urgent laparoscopic surgery [6]. However, the Society of American Gastrointestinal and Endoscopic Surgeons (SAGES) [7] states that laparoscopy can be safely performed during any trimester of pregnancy. Since our patient presented in the second trimester and, after explaining all the risks, chose to proceed with the surgery, we performed the procedure successfully.

Regarding the size of the mass, masses found incidentally during pregnancy in the first trimester that are less than 5 cm in size will resolve on their own by the second trimester. If cysts measuring 5-10 cm persist beyond 16 weeks, they should be carefully monitored with USG, colour Doppler, and, if possible, MRI. For cysts larger than 10 cm, surgical intervention is recommended due to the risk of torsion, malignancy, and labour obstruction in pregnancy [8].

Recent case reports describe asymptomatic large ovarian masses presenting at term [9-11]. In one case, a 25 x 19 cm serous cystadenoma presented with malpresentation (breech) at term, necessitating a cesarean section [9]. Another was an incidental finding during a cesarean section, diagnosed as maternal ascites preoperatively [10]. In the third case [11], a 17 cm cyst was diagnosed at 16 weeks but the patient did not follow up and presented directly at 35 weeks with a 30 cm cyst. She had complaints of shortness of breath, abdominal discomfort, inability to sleep comfortably, and fullness in the abdomen even after eating small amounts of food, starting at 31 weeks. A cesarean section was performed in this case due to a transverse lie. The cyst, a serous cystadenoma, was twisted, but blood supply was restored. Most large ovarian cysts are benign, with the majority being serous cystadenomas, followed by mucinous cystadenomas [12]. We found a single case in the literature involving a second-trimester asymptomatic mucinous cystadenoma measuring 16 x 12 cm, for which a midline vertical laparotomy was performed, and the cyst was removed in toto [13].

Our case is rare because we performed laparoscopic management of a 20 cm cyst at 20 weeks of pregnancy. Although studies comparing laparoscopy and laparotomy for ovarian masses include both prospective randomized and retrospective trials, most of these studies do not involve such large ovarian masses [14-16].

A randomized controlled trial by Chen et al. [14] on 69 patients with asymptomatic adnexal masses at 16 weeks of gestation randomized them to laparoscopy or laparotomy. The mean cyst diameter in these patients was 8 cm. The results showed a shorter hospital stay, less fever and pain postoperatively, and less adhesion at the time of cesarean delivery in the laparoscopy group compared to the laparotomy group. There were no statistically significant differences in the need for cesarean delivery, time of delivery, birth weight, or Apgar scores [14].

Shigemi et al., in a retrospective study of 740 patients, found shorter hospital stays, less blood transfusion, and less operating time in the laparoscopy group compared to the laparotomy group. They also observed a decreased rate of adverse fetal outcomes (miscarriage, preterm birth, and stillbirth) in the laparoscopy group compared to the laparotomy group (0.4% vs. 1.8%, p = 0.01) [15].

In two recent large meta-analyses of ovarian masses (with a mean cyst size of 8.8 cm) in pregnancy, Cagino et al. found that laparoscopy resulted in a shorter hospital stay but there were no differences in blood loss, operation time, miscarriage, or preterm birth between the laparoscopy and laparotomy groups [5]. Ye et al., in their study including 985 patients from nine retrospective studies of ovarian masses in pregnancy, found similar results, except that blood loss was lower, and the odds of preterm labour were 51% lower in the laparoscopy group compared to the laparotomy group [16].

A narrative review by Senarath et al. [17] showed that laparoscopy has a lower thrombotic risk, with management including tocolysis, steroids, and non-teratogenic antibiotics. Although guidelines do not recommend tocolysis, we administered a single shot before surgery for prophylaxis.

For pregnant women requiring laparoscopic surgeries, the best practices can be summarized as [7,18,19]: (i) left lateral decubitus position after the first trimester to prevent aorta compression; (ii) port placement according to uterine size and pathology location; (iii) insufflation pressure of 12 to 15 mm Hg; (iv) screening for venous thromboembolic risk, with mechanical and chemical thromboprophylaxis as necessary, as pregnancy itself is a hypercoagulable state; (v) intraoperative maternal capnography; (vi) fetal heart rate and contraction monitoring both preoperatively and postoperatively; and (vii) antenatal corticosteroids can be considered, but tocolytics, antibiotics, and anti-D immune globulin are not recommended.

## Conclusions

Functional ovarian cysts are common in the first trimester of pregnancy, but if the cyst persists beyond 16 weeks, it becomes a matter of concern. Large ovarian cysts in pregnancy are rare. Even in cases of large benign cysts, greater than 10 cm, laparoscopy is safe during pregnancy when performed by experienced hands with adequate anaesthetic facilities, without adverse effects on the fetus. However, proper case selection is essential. This case scenario has limitations, as we only observed the risk of abortion during hospitalization and up to 21 days after surgery, due to the patient being lost to follow-up. We have not observed any risk of preterm delivery or low birth weight later in the pregnancy. Further studies are needed before this can be recommended as routine practice, to support the future development of treatment protocols in this field, as a single case is insufficient.
